# Attention-Deficit Hyperactivity Disorder and Comorbid Mental Health Conditions Associated with Increased Risk of Injury

**DOI:** 10.1155/2022/2470973

**Published:** 2022-10-14

**Authors:** Ray M. Merrill, Andrew W. Merrill, Miranda Madsen

**Affiliations:** Department of Public Health, College of Life Sciences, Brigham Young University, Provo, Utah, USA

## Abstract

**Background:**

To describe the influence of attention-deficit hyperactivity disorder (ADHD) and comorbid mental health conditions on the risk of selected injuries.

**Methods:**

A retrospective cohort study design was employed using medical claim data from the Deseret Mutual Benefit Administrators (DMBA). Mental health conditions, injury, medication, and demographic data were extracted from claim files for ages 4-64, years 2016-2020.

**Results:**

Approximately 51.8% of individuals with ADHD had one or more comorbid mental health conditions (anxiety [37.0%], depression [29.9%], autism spectrum disorder (ASD) [3.6%], bipolar disorder [4.7%], obsessive compulsive disorder (OCD) [2.4%], schizophrenia [0.9%], and manic disorder [0.2%]). The rate of injury was 1.33 (95% CI 1.27–1.39) for ADHD only versus no ADHD and 1.62 (95% CI 1.56–1.68) for ADHD and comorbid mental health conditions versus no ADHD, after adjusting for age, sex, salary, and year. Cases with ADHD but no comorbid mental health conditions versus no ADHD were at increased risk of each of 12 types of injury. The increased risk was noticeably more pronounced for ADHD cases with one or more comorbid mental health conditions versus no ADHD. The greatest increased risk of injury was among ADHD cases with comorbid schizophrenia, followed by bipolar disorder and OCD. Comorbid autism disorder does not increase the risk of injury, but lowers it. Finally, the number of comorbid mental health conditions among ADHD cases was positively associated with increased injury rates (6% for one, 30% for two, 65% for three, and 129% for four).

**Conclusions:**

ADHD is positively associated with an increased risk of injury. Comorbid mental health conditions further increase the risk of injury among those with ADHD.

## 1. Introduction

The American Psychiatric Association characterizes attention-deficit hyperactivity disorder (ADHD) as “a neurodevelopmental disorder defined by impairing levels of inattention, disorganization, and/or hyperactivity-impulsivity” [1]. Until recently, ADHD has been classified exclusively as a childhood disorder, but scientific literature now recognizes its prevalence across all age groups [2]. In 2016, a reported 8.4% of children (ages 2-17 years) in the U.S. had a current diagnosis of ADHD, and in 2012, an estimated 4.2% of adults (ages 18-64 years) in the U.S. had a current diagnosis of ADHD [3, 4]. Diagnosed ADHD is up to twice as prevalent in males as in females according to the U.S. National Health Interview Survey, but it is suspected that females with ADHD may be underrepresented in this count [4]. ADHD is more prevalent among higher and upper-middle income countries as compared with low- and lower-middle income countries [5]. However, another study involving a Korean National Health Insurance cohort found that children living in decreasing or consistently low-income households compared with mid-high income households had a greater risk of ADHD [6].

ADHD is often diagnosed with at least one other mental, behavioral, or personality disorder, which complicates the clinical picture, assessment, and treatment [7–9]. Because ADHD symptoms usually present themselves before age 7, successful treatment of the disorder may help prevent or lower comorbid mental health conditions that tend to manifest in later ages [7]. The presence of a comorbid illness often changes the clinical presentation, prognosis, and treatment among ADHD cases [10]. Common comorbid problems associated with ADHD include autistic spectrum disorder (ASD), mania, schizophrenia, bipolar disorder, obsessive-compulsive disorder (OCD), depression, anxiety, learning disorders, sleep disorders, oppositional-defiant disorder (ODD), substance use disorders, and personality disorders [7–14]. A systematic review of the literature from 2019 found that bipolar disorder is a common comorbid condition for ADHD, with overlapping symptoms and complex treatment [15].

ASD is an intriguing codiagnosis with ADHD because ASD cases often share some of the core symptoms of ADHD (attention deficit, impulsivity, hyperactivity). However, many of the core symptoms of ASD are not shared with ADHD cases, thus helping to distinguish the two disorders [12]. In a recent review, researchers found up to 70% of individuals with ASD have ADHD symptoms and up to 60% of individuals with ADHD experience the social impairments common in ASD [16]. The authors of this review concluded that ADHD and ASD should be treated as overlapping, yet distinct disorders.

Research has shown that ADHD is positively associated with emergency services, hospitalization, and healthcare claims for injuries [17–22]. A study involving medical claim data in the U.S. showed that for ages 0-64 years, more severe injuries (versus less severe injuries) and an increasing number of injuries were positively association with ADHD [17]. A Dutch study utilizing pharmaceutical records for children and adolescents found that those using ADHD medication were 2.2 (95% CI 1.7-2.9) times more likely to be hospitalized for injuries and 4.8 (95% CI 1.4-16.9) times more likely if they had concomitant psychotropic drugs [18]. In a study involving medical and pharmaceutical claim data for enrollees in a large insurance database in the U.S., increased risk of injury among ADHD cases became significantly more pronounced for those with comorbid psychosis, neurotic, or personality disorder [23]. The purpose of the current study is to identify any and specific types of injury rates according to ADHD with and without comorbid mental health conditions. The study extends previous research by involving selected comorbid conditions not previously considered in ADHD-injury correlation analysis based on medical claim data. The relative contribution of these comorbid conditions and the number of comorbid conditions on injury will be considered.

The rate of comorbid mental health conditions with ADHD and ADHD associated injury will be assessed according to age, sex, and salary. Twelve specific types of injury rates will be compared between no ADHD, ADHD only, and ADHD and comorbid mental health conditions.

## 2. Materials and Methods

### 2.1. Study Population

The study population involves individuals receiving health insurance from a large company called the Deseret Mutual Benefit Administrator (DMBA). The company was established in 1970 to provide health insurance and retirement income to employees and their families of the Church of Jesus Christ of Latter-day Saints. Electronic claim data were available for the years 2016 through 2020. Enrollees during this time resided in Utah (73%), Idaho (10%), other mountain states (4%), pacific states (8%), central states (4%), and eastern states (1%).

Each year, the cohort consists of approximately 27% contract holders, 21% spouses, 48% dependent children, and 4% other (e.g., married child, stepchild, and disabled dependent). Among contract holders, 34% work in the Church Education System, seminaries, and institutes; 31% work as manual laborers; 10% work in other companies; 6% are retired; and the remaining 19% work in other capacities. Employee retention is about 92% (80% in ages 18-29, 95% in ages 30-64, and 76% in ages 65 or older) from year to year. The high percentage of contract holders who are no longer insured through DMBA in the ages 65 or older is because they become eligible for Medicare, a program under the U.S. Social Security Administration that reimburses hospitals and physicians for medical care provided to qualified individuals ages 65 or older.

### 2.2. Data Collection

We examined employee eligibility data and matched automated claims records from January 1, 2016, to December 31, 2020. The International Classification of Diseases, Tenth Revision, Clinical Modification (ICD-10-CM) codes were used to classify injuries and ADHD [24]. Records were linked using a common identifying number. The database was deidentified according to Health Insurance Portability and Accountability Act (HIPAA) guidelines. Ethical approval and informed consent to participate were waived by the authors' institutional review board because the data were anonymized before assessment (IRB2021-157).

The ICD-10-CM codes used to classify injuries were S00-S09 (head), S10-S19 (neck), S20-S29 (thorax), S30-S39 (Abdomen, lower back, lumbar spine, pelvis, and external genitals), S40-S49 (shoulder and upper arm), S50-S59 (elbow and forearm), S60-S69 (wrist, hand, and fingers), S70-S79 (hip and thigh), S80-S89 (knee and lower leg), S90-S99 (ankle and foot), T20-T28 and T30-T32 (burns and corrosions), and T36-T50 (poisoning).

Individuals with an ICD-10-CM code of F90 were suspected to have ADHD. However, as with most insurance claims, the DMBA claims do not differentiate between a visit where ADHD is suspected and one where a physician diagnosis is made. For this reason, persons were defined as having ADHD if they (a) had two or more visits with an ADHD diagnosis, (b) had a prescription of a drug used to treat ADHD, or (c) had a combination of diagnosis and ADHD drug. Drugs used to treat ADHD included nonstimulants (atomoxetine, guanfacine, and Kapvay) and stimulants (amphetamine, methylphenidate, and dexmethylphenidate). Other mental health disorders considered in this study were anxiety (F40, F1), depression (F32, F33), bipolar disorder (F31), obsessive compulsive disorder (OCD) (F42), schizophrenia (F20–F29), and autism (F840).

Analyses were limited to ages 4 through 64 years. Numbers of enrollees in the DMBA database in this age range were 74,169 in 2016, 76,063 in 2017, 78,302 in 2018, 75,505 in 2019, and 78,449 in 2020. Numbers classified with ADHD in these years were 2,936 (4.0%), 3,199 (4.2%), 3,564 (4.6%), 3,417 (4.5%), and 3708 (4.7%), respectively. A relatively small number of individuals had a single ICD-10-CM code for ADHD and did not receive one of the medication schemes listed above for treating ADHD: 154 in 2016, 125 in 2017, 123 in 2018, 137 in 2019, and 181 in 2020. For these individuals, it appears that the initial workup for ADHD did not confirm the disorder. Hence, they were not included in the ADHD group.

The rate of ADHD consisted of all individuals classified with ADHD in a given year divided by the number of enrollees in that year. If an injury occurred in the same year as a diagnosis with ADHD, we assumed that ADHD influenced the incidence of that injury. Multiple injuries were possible and included in the analyses per year. For example, if a person experienced a head injury and a burn in the same year, they were included in the rate calculation for both head injury and for a burn. If any type of injury (see list above) occurred in a given year, then that person was included in an overall injury rate calculation.

### 2.3. Statistical Techniques

The rate of ADHD (per 100) was presented according to age, sex, salary (of contract holder), and year and evaluated for statistical significance using the chi-square test. Poisson regression was used to compute rate ratios, adjusted for age, sex, salary, and year. Rate ratios measured the association between ADHD status (yes vs. no) and the demographic variables, with interaction terms evaluated. Rate ratios also measured the association between ADHD status (yes vs. no) and each of the other selected mental health conditions, with interaction terms assessed in each model. Analyses then considered how injuries might be influenced by ADHD and comorbid conditions. Specifically, rate ratios measured the association between ADHD status (no ADHD, ADHD only, ADHD and comorbid mental illness) and the presence (yes vs. no) of each of the 14 types of injury; any injury (yes vs. no) among ADHD cases and each of the comorbid mental health conditions (yes vs. no); and any injury (yes vs. no) among ADHD cases and the number of comorbid mental health conditions. In models assessing the rate of injury among ADHD cases and each of the comorbid conditions, interaction terms were included and evaluated. Interaction terms were assessed for ignificance using the Wald chi-square test. Adjusted rate ratios were presented along with their corresponding 95% confidence intervals. Confidence intervals not containing 1 were statistically significant at the 0.05 level. Two-sided tests of significance were used, based on the 0.05 level. Statistical analyses were derived from Statistical Analysis System (SAS) software, version 9.4 (SAS Institute Inc., Cary, NC, USA, 2012).

## 3. Results

During 2016-2020, the average number of DMBA enrollees ages 4-64 each year was 76,498 (49.2% male and 50.8% female). Mean age each year was 30.8 (SD = 18.0). The average number with ADHD each year was 3,365 (4.4%).

### 3.1. Description of ADHD Cases

ADHD significantly varies according to age, sex, salary, and year (Table 1). The rate of ADHD is more common in the age groups 10-14 and 15-19 and then decreases in subsequent age groups. The rate of ADHD is about 46% more common in males than females and tends to increase with salary and year.

**Figure 1:**
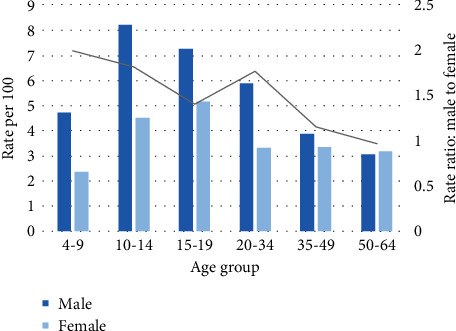
Rate of ADHD according to age and sex.

The rate of ADHD for males compared with females significantly varies across age groups (Figure 1). For the age groups 4-9, 10-14, 15-19, 20-34, and 35-49, the rate of ADHD is approximately 100%, 82%, 40%, 77%, and 16% greater in males than females, respectively. In contrast, in the age group 50-64, the rate of ADHD is approximately 3% lower in males than females.

### 3.2. Associations between ADHD and Other Mental Health Conditions

Among ADHD cases, 51.8% had one or more other mental health conditions. The distribution of ADHD and comorbid mental health conditions is shown in Table 2. Among ADHD cases, 37.0% also had anxiety, 29.9% also had depression, 4.7% also had bipolar disorder, and so on. Those with ADHD compared to those without the disorder were significantly more likely to experience each of the other selected mental health conditions. For example, their increased rate was 13.7 times more likely for autism, 10.2 times more likely for manic disorder, 9.4 times more likely for bipolar disorder, and from 4.3 to 5.8 times more likely for the other disorders, after adjusting for age, sex, salary, and year.

**Figure 2:**
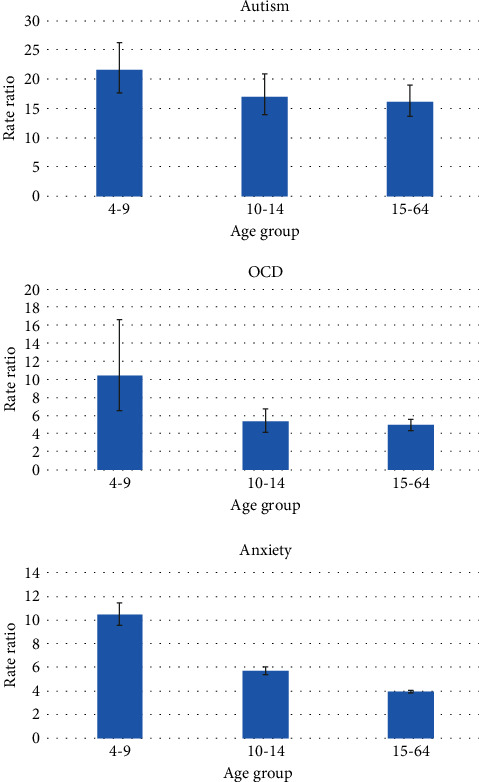
Rate of selected mental health conditions by ADHD case status and age.

Higher rates of each of the mental health conditions (yes vs. no) among those with ADHD (vs. otherwise) significantly varied according to age. Higher rates for each of the mental health conditions are significantly greater in ages 4-9, followed by 10-14, and then 15-64. More specific age groups in the later ages were combined into the last age group because the rate ratios for each mental health condition were similar (data not shown). To illustrate how the rate ratios are associated with age, the increased rate of autism, OCD, and anxiety for those with ADHD versus otherwise are shown in Figure 2.

The greater rate of depression for ADHD cases was significantly more pronounced in males than females (5.05 [95% CI 4.85–5.24] vs. 4.12 [95% CI 3.98–4.26]). There was inconclusive evidence of the greater positive association between ADHD and depression in males compared with females in ages 4-14 years, but there was in ages years 15-64: (4.95 [95% CI 4.75–5.16] for males vs. 3.83 [95% CI 3.70–3.97] for females).

### 3.3. Rates of Injury by ADHD Status and Comorbid Mental Health Conditions

The rate of injury in ADHD cases without comorbid mental health conditions is 19.0%, in ADHD cases with comorbid mental health conditions is 22.7%, and in non-ADHD cases is 13.7%. The rate of injury (yes vs. no) for ADHD cases without comorbid mental health conditions versus those without ADHD is 1.39 (95% CI 1.33–1.45), and those with ADHD and comorbid mental health conditions versus those without ADHD is 1.66 (95% CI 1.60–1.73). Corresponding rate ratios adjusted for age, sex, salary, and year are 1.33 (95% CI 1.27–1.39) and 1.62 (95% CI 1.56–1.68), respectively.

ADHD cases have significantly higher rates of several selected types of injury, after adjusting for age, sex, salary, and year (Table 3). ADHD cases without comorbid mental health conditions compared with no ADHD cases have significantly higher rates for each of the twelve types of injury. The rate of injuries for ADHD cases with comorbid mental health conditions compared with no ADHD cases is consistently greater than for ADHD cases without comorbid mental health conditions. This is especially true for poisoning, burns, and head injuries.

For no ADHD, ADHD only, and ADHD with one or more comorbid mental health conditions, the rates (per 100) are 0.30, 0.54, and 2.26 for poisoning, 0.14, 0.26, and 0.36 for burns, and 2.02, 3.34, and 4.24 for head injuries, respectively. The rate ratio for ADHD with one or more comorbid mental health conditions versus ADHD only is 4.19 for poisoning, 1.38 for burns, and 1.27 for head injuries. Rates of poisoning, burns, and head injuries were 41.10 (95% CI 33.69-50.14), 10.25 (95% CI 5.51-19.07), and 3.14 (95% CI 2.36-4.18) for schizophrenia cases, respectively. Corresponding rate ratios measuring the association between poisoning, burns, and head injuries and bipolar disorder are lower (15.88 [95% CI 13.36-18.87], 2.28 [95% CI 1.18-4.40], and 2.43 [95% CI 2.06-2.86]). Other rate ratios measuring the association between poisoning, burns, and head injuries and OCD or depression or anxiety are even lower, albeit positively significant (data not shown).

Rates of injury among ADHD cases increases with comorbid schizophrenia, bipolar disorder, OCD, depression, and anxiety (Table 4). Among these comorbid conditions, the greatest increased rate is among individuals with schizophrenia, and the lowest increased rate is among individuals with anxiety. Schizophrenia is most strongly associated with poisoning, burns, elbow and forearm, and head injuries; bipolar disorder is most strongly associated with poisoning, thorax, head, and burns; OCD is most strongly associated with poisoning, hip and thigh, thorax, and abdomen and groin; depression is most strongly associated with poisoning, burns, neck, and thorax; and anxiety is most strongly associated with poisoning, neck, burns, and thorax (data not shown). Comorbid autism may lower the rate of injury.

**Figure 3:**
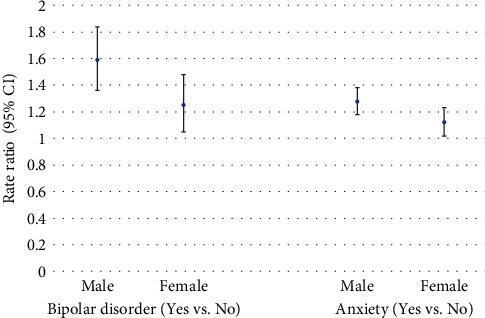
Increased rate of injury for comorbid bipolar disorder and anxiety among ADHD cases by sex.

The increased rate of injury for selected comorbid mental health conditions (schizophrenia, bipolar disorder, OCD, depression, and anxiety) among ADHD cases did not significantly vary according to age group. However, the increased rate of injury for ADHD cases with comorbid bipolar disorder or anxiety significantly varied by sex (Figure 3). Specifically, the rate of injury for ADHD cases with comorbid bipolar disorder (vs. no bipolar disorder) was 59% higher for males but 25% higher for females (*p* = 0.0410). The rate of injury for ADHD cases with comorbid anxiety (vs. no anxiety) was 28% higher for males but 12% higher for females (*p* = 0.0342).

### 3.4. Injury Rates in ADHD Cases by Number of Comorbid Mental Health Conditions

The number of comorbid mental health conditions among ADHD cases is positively associated with increased risk of injury (Table 5). For ADHD cases with one, two, three, or four additional comorbid mental health conditions, the rate of injury goes up 6%, 30%, 65%, and 129%, respectively. The increasing trend is statistically significant and does not vary according to age or sex.

## 4. Discussion

This study showed the incidence rate of ADHD by selected variables, being greatest in the age groups 10-14 and 15-19, among males, and where the contract holder's salary was $100,000 or more. These results are consistent with other studies [3–6]. The current study also found that the greater rate of ADHD among males than females decreased across the age span until, by ages 50-64, an increased rate was no longer seen. This is consistent with findings in other studies that observed a negative relationship between ADHD and age [17, 23].

The majority of ADHD cases have comorbid mental health problems. Other studies have also observed a high level of comorbid mental illness among ADHD cases [7–16]. Because depression and anxiety are comparatively more common among the mental health problems, a higher percentage of ADHD cases had these comorbid conditions. However, ADHD had the strongest rate association with autism. Research has shown a strong correlation between these two mental health problems [16]. The greater rate of other mental health problems for ADHD cases was more pronounced in the youngest age group 4-9 years, followed by 10-14. Understanding the causal direction here is complex, but in a child's early education, mental health problems in general may lead to ADHD behavior and subsequent diagnosis. In addition, the greater rate of depression among ADHD cases was more pronounced in males than females, but only in ages 15 and older. Because of the religious culture this study represents, it may be that males feel a greater responsibility to be the primary breadwinner, but given their disorder, are more likely to experience depression.

Consistent with findings in the current study, previous research has observed an increased rate of injury among ADHD cases [17–22]. We observed that this increased rate occurred in each injury classification considered, especially poisoning. Comorbid mental health problems make ADHD cases even more prone to accidental poisoning. Other research has also found that ADHD is associated with increased rate of poisoning, more so when comorbid mental disorders are present, and that the relative rate of poisoning was significantly greater than physical injuries [25]. In other words, specific characteristics of the disorder such as inattention and impulsivity appear to be more strongly associated with poisoning.

We further observed that the significantly increased rate of injury among ADHD cases was 1.33 (95% CI 1.27–1.39) for ADHD alone and 1.62 (95% CI 1.56–1.68) for ADHD and comental health problems, after adjusting for age, sex, salary, and year. This is also consistent with previous research [23]. However, that previous study did not consider the extent of comorbid mental health problems as assessed in the current study.

The rate of injury among ADHD cases significantly increased with comorbid schizophrenia, bipolar disorder, OCD, depression, and anxiety. Hence, features of the disorder, which increase the rate of injuries, are compounded with the comorbidity of these other mental health conditions. The increased rate of injury was most pronounced in ADHD cases with comorbid schizophrenia, and the increased rate was least pronounced for ADHD cases with comorbid anxiety. Schizophrenia was more strongly associated with poisoning and burns, followed by head injuries, than the other mental health conditions.

When an ADHD case also had autism, their rate of injury decreased. Understanding which autism symptoms may be protective against injury deserves further investigation. In addition, the increased rate of injury for bipolar disorder and anxiety varied by sex, being more pronounced for males than females. Our data limits us from being able to explain these findings, especially given that injuries like traumatic brain injury may both be the cause and the result of these mental health disorders, at different levels for males and females.

The number of comorbid mental health conditions among ADHD cases is positively associated with increased risk of injury. This is consistent with results from previous research [17]. It may be that ADHD cases with additional forms of mental illness have more severe ADHD type symptoms, like inattention and impulsivity, which correlate with an increased risk of injury.

### 4.1. Limitations

The study was based on medical and pharmaceutical claims from contract holders and their dependents in the DMBA databased during 2016 through 2020, so no selection of subgroups that might have resulted in bias was present. However, generalization of the results is limited because the contract holders (27% of enrollees) are employees, so that they and their dependents are less likely to represent lower income individuals in the general U.S. population. Unemployed, lower-income families may represent different levels of ADHD. In addition, the specific religious preference of the individuals in this study was not available, but the nature of the database and known job types suggests that most were members of the Church of Jesus Christ of Latter-day Saints. This religious group is known for their relatively low use of tobacco smoking, alcohol drinking, and illicit drug use [26–31]. Research has shown that ADHD is positively associated with tobacco, alcohol, and illicit drug use [32]. If use of these substances increases the rate of injury, then the current results would underestimate the association between ADHD and injury for the general population.

All contract holders, regardless of salary, were eligible to receive comprehensive insurance coverage. An ADHD diagnosis would not lead to losing insurance so making selective underreporting of ADHD claims for this reason unlikely. However, it may be that some ADHD cases were not identified by the criteria used in this study. Incorrect diagnosis and treatment of ADHD is also possible. The available data did not allow us to identify the level of under or over reporting of ADHD due to these potential influences.

A small number of individuals who visited the doctor for an ADHD workup failed to receive a confirmation of the disorder. We assumed this because they neither received medication nor a follow-up visit for ADHD. Yet, it is possible that some of these people did have ADHD, and including them in the non-ADHD group could have created a conservative positive relationship between ADHD and injury. Furthermore, the fact that some minor injuries may not have been included in the claim database could have dampened the observed association between ADHD and injury.

## 5. Conclusions

The majority of ADHD cases have one or more comorbid mental health conditions. ADHD is significantly associated with increased risk of autism, manic disorder, bipolar disorder, schizophrenia, OCD, anxiety, and depression (from largest to smallest increased rates). Increased rates of these conditions in ADHD cases is the highest in ages 4-9 and lowest in ages 15-64. Greater risk of depression for ADHD cases is more pronounced in males than females but only in ages 15-64.

While ADHD cases have an increased risk of 12 general types of injury, the addition of comorbid mental health problems significantly increases the already increased risk, especially for poisoning. The combination of ADHD and each of the mental health conditions considered increased the risk of injury, irrespective of age, with the addition of schizophrenia, bipolar disorder, or OCD most dangerous in terms of increasing the risk of injury. When ADHD was combined with bipolar disorder or anxiety, the increased risk was more pronounced for males versus females. ADHD cases with autism have significantly lower risk of injury than ADHD cases without autism. The number of comorbid mental health conditions in ADHD cases increases the risk of injury, irrespective of age and sex.

## Figures and Tables

**Table 1:** Attention-deficit hyperactive disorder (ADHD) in the Deseret Mutual Benefit Administration (DMBA) population according to selected variables, 2016-2020.

	ADHD no.	ADHD %	Chi-square *P* value	Rate ratio^†^	95% confidence interval^†^
Age (years)					
4-9	1630	3.60	<0.0001	1.00	—
10-14	2862	6.46		1.77	1.67–1.88
15-19	2952	6.25		1.71	1.61–1.82
20-34	4251	4.58		1.32	1.25–1.40
35-49	2627	3.60		1.01	0.95–1.07
50-64	2502	3.13		0.90	0.85–0.96
Sex					
Female	6884	3.55	<0.0001	1.00	—
Male	9940	5.28		1.46	1.42–1.51
Salary (of contract holder)					
0-39,999	1565	3.29	<0.0001	1.00	—
40,000-69,999	3789	4.40		1.33	1.23–1.38
70,099-99,999	4502	4.47		1.30	1.23–1.38
100,000+	6313	5.01		1.44	1.36–1.52
Unknown	655	2.96		0.91	0.83–0.99
Year					
2016	2936	3.96	<0.0001	1.00	—
2017	3199	4.21		1.06	1.01–1.11
2018	3564	4.55		1.14	1.08–1.19
2019	3417	4.53		1.15	1.10–1.21
2020	3708	4.73		1.17	1.12–1.23

^†^Adjusted for the other variables in the model.

**Table 2:** Rate of other types of mental health problems among ADHD cases compared with non-ADHD cases.

ADHD+	No.	%	Rate ratio^†^	95% confidence interval^†^
Anxiety	6228	37.02	4.34	4.24–4.43
Depression	5029	29.89	4.31	4.20–4.42
Bipolar disorder	784	4.66	9.37	8.62–10.18
Autism	609	3.62	13.68	12.28–15.24
OCD	400	2.38	4.45	4.00–4.96
Schizophrenia	148	0.88	5.82	4.84–7.01
Manic	25	0.15	10.16	6.22–16.60

^†^Adjusted for age, sex, salary, and year.

**Table 3:** Rate of selected types of injury for ADHD cases compared with non-ADHD cases.

					ADHD only		ADHD plus one or more other mental health problems	
Injuries to the …		No.	%	No ADHD	Rate ratio^†^	95% confidence interval^†^	Rate ratio†	95% confidence interval^†^
Head	S00-S09	8027	2.10	1.00	1.47	1.31–1.66	1.99	1.79–2.20
Neck	S00-S09	4114	1.08	1.00	1.45	1.21–1.75	1.66	1.41–1.95
Thorax	S10-S19	4043	1.06	1.00	1.68	1.41–1.99	1.85	1.58–2.17
Abdomen and groin^‡^	S20-S29	6194	1.62	1.00	1.45	1.25–1.68	1.50	1.31–1.73
Shoulder and upper arm	S30-S39	5478	1.43	1.00	1.43	1.23–1.66	1.62	1.41–1.87
Elbow and forearm	S40-S49	4158	1.09	1.00	1.35	1.14–1.60	1.58	1.35–1.85
Wrist, hand and fingers	S50-S59	11081	2.90	1.00	1.51	1.37–1.67	1.72	1.57–1.89
Hip and thigh	S60-S69	2452	0.64	1.00	1.32	1.04–1.68	1.58	1.28–1.95
Knee and lower leg	S70-S79	10093	2.64	1.00	1.19	1.06–1.35	1.50	1.35–1.66
Ankle and foot	S80-S89	10885	2.85	1.00	1.18	1.05–1.33	1.54	1.40–1.70
Burns	S90-S99	565	0.15	1.00	1.87	1.20–2.90	2.52	1.75–3.62
Poisoning	T20-T28 T30-T32	1351	0.35	1.00	2.00	1.48–2.71	7.55	6.47–8.81

^†^Adjusted for age, sex, salary, and year. Other mental health problems refer to one or more of the problems listed in Table 2. ^‡^Abdomen, lower back, lumbar spine, pelvis, and external genitals.

**Table 4:** Rate of injury among ADHD cases according to whether other comorbid mental health conditions exist.

	Yes %	No%	Rate ratio^†^	95% confidence interval^†^	Rate ratio^‡^	95% confidence interval^‡^
Schizophrenia	41.89	20.72	1.94	1.58–2.38	1.74	1.25–2.41
Bipolar disorder	29.08	20.51	1.42	1.27–1.60	1.31	1.15–1.50
OCD	27.50	20.75	1.35	1.14–1.59	1.24	1.03–1.51
Depression	24.28	19.47	1.24	1.17–1.33	1.16	1.08–1.25
Anxiety	23.47	19.40	1.22	1.15–1.30	1.14	1.06–1.22
Autism	18.39	21.01	0.87	0.73–1.03	0.79	0.65–0.96
Manic disorder	12.00	20.92	0.60	0.21–1.69	0.52	0.15–1.72

^†^Adjusted for age, sex, salary, and year. ^‡^Adjusted for age, sex, salary, year, and the other mental health variables in the table.

**Table 5:** Rate of injury among ADHD cases according to the number of comorbid mental health problems.

	No.	%	Injury rates %	Rate ratio^†^	95% confidence interval^†^
ADHD only	8,118	48.25	18.99	1.00	
ADHD +1	4,850	28.83	20.21	1.06	0.99–1.14
ADHD +2	3,269	19.43	24.53	1.30	1.20–1.40
ADHD +3	517	3.07	31.72	1.65	1.43–1.90
ADHD +4 or more	70	0.42	42.86	2.29	1.68–3.12

^†^Adjusted for age, sex, salary, and year.

## Data Availability

The datasets generated and analyzed during the current study are not publicly available due to confidentiality restrictions but are available in a deidentified and aggregated format from the corresponding author on reasonable request.

## Abbreviations

ADHD: Attention-deficit hyperactivity disorder
OCD: Obsessive compulsive disorder
DMBA: Deseret Mutual Benefit Administrators
ICD-10-CM: International Classification of Diseases, Tenth Revision, Clinical Modification.
